# Systematic Approaches towards the Development of Host-Directed Antiviral Therapeutics

**DOI:** 10.3390/ijms12064027

**Published:** 2011-06-15

**Authors:** Andrew Prussia, Pahk Thepchatri, James P. Snyder, Richard K. Plemper

**Affiliations:** 1 Department of Chemistry, Emory University, Atlanta, GA 30322, USA; E-Mails: aprussi@emory.edu (A.P.); jsnyder@emory.edu (J.P.S.); 2 Emory Institute for Drug Discovery (EIDD), Emory University, Atlanta, GA 30322, USA; 3 Department of Pediatrics, Emory University, Atlanta, GA 30322, USA; 4 Children’s Healthcare of Atlanta, Atlanta, GA 30322, USA

**Keywords:** genome-wide screening, pathway analysis, HIV, Influenza virus, bioinformatics, antiviral, target identification, RNAi, siRNA

## Abstract

Since the onset of antiviral therapy, viral resistance has compromised the clinical value of small-molecule drugs targeting pathogen components. As intracellular parasites, viruses complete their life cycle by hijacking a multitude of host-factors. Aiming at the latter rather than the pathogen directly, host-directed antiviral therapy has emerged as a concept to counteract evolution of viral resistance and develop broad-spectrum drug classes. This approach is propelled by bioinformatics analysis of genome-wide screens that greatly enhance insights into the complex network of host-pathogen interactions and generate a shortlist of potential gene targets from a multitude of candidates, thus setting the stage for a new era of rational identification of drug targets for host-directed antiviral therapies. With particular emphasis on human immunodeficiency virus and influenza virus, two major human pathogens, we review screens employed to elucidate host-pathogen interactions and discuss the state of database ontology approaches applicable to defining a therapeutic endpoint. The value of this strategy for drug discovery is evaluated, and perspectives for bioinformatics-driven hit identification are outlined.

## 1. Introduction

With few exceptions, therapeutic approaches to combat infectious diseases have focused in the past decades on targeting unique components or enzymes of viral, bacterial and parasitic origin. It has now become perfectly evident that this traditional, pathogen-directed strategy, while highly successful in numerous cases [1], is inherently compromised by the rapid emergence of resistance or, increasingly, pre-existing pathogen resistance to individual drugs. For example, the efficacy of current neuraminidase inhibitors for the treatment of pandemic swine origin influenza H1N1 isolates is increasingly compromised by the appearance of viral strains with pre-existing resistance in the field [2–4]. Drug-resistant variants have likewise propelled the re-emergence of highly pathogenic bacteria strains such as Mycobacterium tuberculosis decades after they were considered contained [5,6].

Prompted largely by the onset of the global HIV epidemic, the development of combination therapies based on drugs with distinct individual resistance profiles has considerably heightened the barrier against the development of pathogen resistance and frequently boosted the effectiveness of pathogen-directed therapeutics through synergistic effects [7]. Despite these successes, however, combination therapies have not conceptually addressed the problem of pathogen resistance and multidrug resistant variants that emerge frequently in clinical settings and cohorts of highly therapy-experienced patients. Developing new generations of inhibitors that are inherently prohibitive of the rapid development of resistance will rather require a new and complementary paradigm for drug discovery.

### 1.1. Host-Directed Antivirals, a New Paradigm for Management of Viral Diseases

Of the strategies entertained towards this goal, targeting host factors that are essential for the pathogen life cycle, rather than pathogen components directly, has recently received increasing attention [8–10]. While all pathogenic microbes experience interactions with their host organisms, viruses as obligatory parasites are directly dependent upon their host cell environment for replication, protein expression and assembly of progeny particles. It is anticipated that blocking one or more of these critical host components or cellular pathways will be resilient to the rapid development of viral resistance, since individual point mutations in viral components are unlikely to compensate for the loss of an essential host factor.

Indeed, the currently available data for the experimental use of host cyclin-dependent kinase (CDK) inhibitors to block HSV-1 and HIV-1 replication, for instance, has revealed a remarkably reduced frequency of viral escape from inhibition in tissue culture settings [11,12]. In contrast, single point mutations in viral components are fully sufficient to abrogate high-affinity binding of pathogendirected antivirals as demonstrated by numerous studies investigating the molecular mechanism of viral drug resistance [1]. Given that replication of related viral pathogens frequently depends on overlapping host cell pathways, host-directed antiviral strategies have high potential to move beyond the one-bug one-drug paradigm by broadening the pathogen target range of a chemical agent.

### 1.2. Identification of Suitable Targets for Host-Directed Antivirals

From a therapeutic perspective, the intricate and complex network of virus-host interactions yields a multitude of potential cellular targets for host-directed antivirals. In addition to the aforementioned role of CDK host protein kinases in HSV-1 and HIV-1 replication [11,12], some examples include regulatory kinases of the Abl [13] and Src [14] tyrosine kinase families, inhibition of which blocks poxvirus motility and maturation of West Nile virus particles, respectively. The Raf/MEK/ERK kinases of the mitogen-activated protein kinase (MAPK) cascades [15], when inhibited, induce nuclear retention of the influenza virus ribonucleoprotein complexes [16], preventing their export and ultimately influenza virion assembly. A further example includes inhibition of COX-2, a component of the eicosanoid biosynthesis pathway, which reduces yields of human cytomegalovirus progeny virus [17].

Clearly, the most desirable host target is essential for completion of the pathogen life cycle under investigation but at least temporarily dispensable for host cell survival, thus supporting the prospect that successful inhibition will combine a potent antiviral effect with manageable toxicity. Nevertheless, targeting host factors carries an inherently higher potential for undesirable drug-induced side effects than pathogen-directed antiviral therapies, particularly when the latter is highly selective. While host-directed therapies are being explored for the treatment of some major chronic viral infections such as HSV-1 and HIV-1 [11,12], they appear predestined for the therapy of infections by pathogens predominantly associated with severe acute disease, since anticipated treatment times and concomitant host exposure to the drug remain limited. On the other hand, chronic infections are conceivably treatable by host-protein targets where more than one gene pathway regulates the condition. In some such cases, sequential application of host-gene modifiers could control disease progression without undue side effects associated with chronic application of an anti-viral drug.

Hit candidates for host-directed drug development programs have resulted from a diverse set of experimental approaches. These can be grouped largely into knowledge-driven direct identification of individual targets, automated screening of chemical diversity sets with protocols specifically designed for the discovery of host-directed hits, and systems-wide screens for host factors essential for pathogen replication.

The evolving understanding of critical host-pathogen interactions through molecular virology research of individual viral families has made possible the direct selection of candidate host targets. With an arsenal of approved and experimental therapeutics for inhibition of many cellular pathways at hand, identified candidate targets may in many cases be immediately testable through repurposing of known drugs or commercially available experimental compounds with known bioactivity. The demonstration that Gleevec (Imatinib mesylate), an Abl tyrosine kinase inhibitor licensed for the treatment of several cancer forms [18], is a poxvirus blocker [13] and the repurposing of the MEK kinase inhibitor U0126 to block the Raf/MEK/ERK cascade for influenza virus inhibition [16,19] serve as cases in point. While the former originated from the insight that efficient vaccinia virus spread requires phosphorylation of the viral A36R protein by Abl and Src family tyrosine kinases [20,21], the latter was triggered by the observation that influenza virus infection induces the activation of MAPK family members [16,21].

The availability of large robotic capacities in both corporate and academic settings combined with the rapid design and production of small-molecule compound libraries in the past two decades has accelerated the pace of discovery of novel drug candidates via high-throughput screening exercises. Applied to the identification of antiviral hits with a host-directed activity profile, for instance, it should be feasible to derive suitable screening protocols based on the hypothesis that host-directed candidates will likely show some cellular interference and return a broadened pathogen target range. While the former will translate into a lower primary screening score represented by the selectivity index (CC_50_/EC_50_), the latter should result in efficient inhibition not only of the screening agent but also of pathogens of related viral families when assessed in counter-screening assays. When we explored the general feasibility of this approach conceptually using a ~140,000-entry diversity set, several chemical compound classes were identified that efficiently blocked replication of a panel of distinct members of the myxovirus families [22]. Significantly, a subset of these revealed a host-directed activity profile in secondary assays and counter-screening exercises.

To determine the molecular target of host-directed compounds identified through screening of chemical libraries, a combination of traditional mechanism of action studies, genomics (*i.e.*, gene microarrays), and/or proteomics (*i.e*., protein profiling) studies is conceivable. Target identification not only sets the stage for possible knowledge-based scaffold optimization through rationale design in conjunction with hit-to-lead chemistry or repurposing of known inhibitors with identical target profile, but also contributes to further elucidating critical pathogen host interactions and, thus, basic insight into pathogen biology.

A second unbiased approach for host-target identification centers on screens for host factors directly interacting with viral components or required for successful completion of the viral life cycle. Of these, avidity-based extracellular interaction screens (AVEXIS) of protein-protein contacts [23] and yeast two-hybrid screens [24,25] appear promising, although they remain inherently limited to specific pathogen factors selected as “baits”. In contrast, loss-of-function screens based on aptamers [26,27] or antisense RNA interference [28–31] and gain-of-function approaches utilizing expression libraries [32,33] afford a systems-wide view of host-factors essential for pathogen replication or boosting pathogen success, respectively. In recent years, groundbreaking “loss-of-function” antisense screens were carried out for major viral pathogens including influenza virus [34,35], human immunodeficiency virus (HIV) [36–39] West Nile virus [40] and hepatitis C virus [41]. These have transformed our understanding of the virus-host interplay.

**Figure 1:**
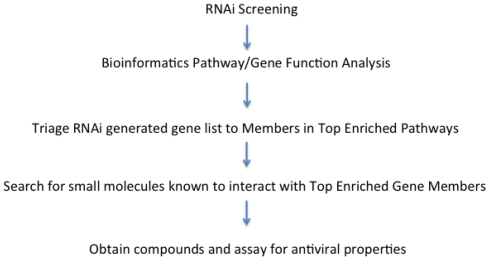
RNAi-Based Lead Identification Workflow. Aberrant expressed genes identified in the RNAi screen are categorized into clusters based on biological function. Members of the largest clusters are literature and database-mined for known small molecule modulators. Candidate inhibitors are subjected to biotesting for hit confirmation. This review focuses on the ability of bioinformatics methods to identify potential medicinal lead compounds.

Surprisingly, however, independent large-scale screens directed at influenza virus, for instance, returned very little redundancy for essential host factors identified. This suggests that the screening efforts still lack saturation, and that cross-study bioinformatics efforts for data mining would benefit significantly by commencing on a host cell pathway rather than at the individual protein level. When a set of identified pathways essential for virus replication emerges through bioinformatics selection, individual components can be subjected to secondary screens using known drugs or experimental inhibitors to short-list desirable individual targets. A proposed workflow for a bioinformatics lead-identification process is displayed in Figure 1. In the following, we will discuss in detail the current state of data mining approaches and how they have been, or could be, applied to screening results reported for HIV-1 and influenza A viruses.

## 2. Methods to Analyze and Process Viral Host Factors Identified as Hits

As with the output of chemical genomic screens, genome-wide association studies (GWAS) require bioinformatics support to analyze high density data points and highlight the important gene or gene sets responsible for the phenotype under investigation. False positives need attention beyond the high throughput experiment when choosing targets for further study. These data points often arise when certain genes have been incorrectly identified due to off-target effects associated with siRNA inhibition. In one case, a single siRNA reportedly perturbed the expression of over 300 genes [42]. The potential quantity of false positives generated from a single RNAi screening experiment becomes alarming, when a single RNAi can potentially affect a large subset of gene expressions. It has been hypothesized that clustering methods such as pathway analysis and gene functional analysis are a possible means to discard false positives and highlight true positives, since they readily generate a biological interpretation of a high throughput result. A number of reviews have summarized the state of genetic analysis [43–51]. In this article, we outline the GWAS approach to antiviral identification and then highlight how such methods have been used to probe host-virus interactions.

Currently, several curated databases in the public domain detail known gene product associations (see Table 1 for examples). As will be illustrated in the following, different databases may return a diverse set of answers even when identical RNAi screening results are used as input.

**Figure 2:**
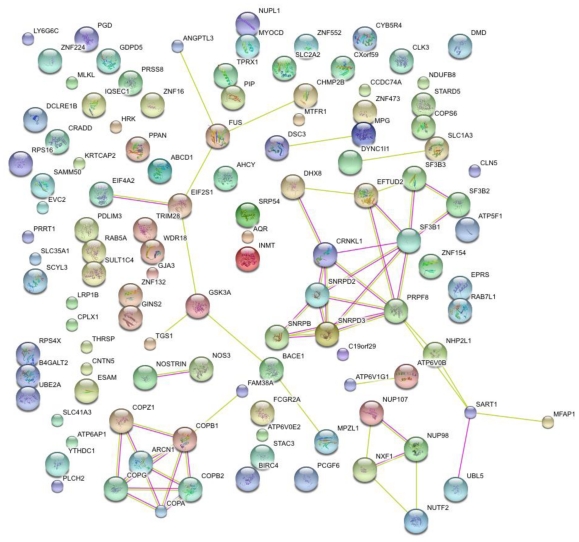
Network Association Map of RNAi screening results generated by the Brass *et al*. influenza virus infection. The list of perturbed host cell genes were complied in STRING and illustrated as nodes above. Lines between different nodes (edges) represent protein interactions that are either known experimentally (purple) or predicted computationally (yellow). Significant nodes such as those shown around COPA and CRNLK1 suggest these pathways to be critical for the viral life cycle.

Application of GWAS frequently employs graphical networks displaying interconnected genes as exemplified in Figure 2. The ability to interconnect and cluster genes by known function forgoes the need to verify every target that is generated by an RNAi screen through a multitude of single biological experiments. Topological representations of biological function identify enriched regions of perturbed gene expression and their relevant cellular operations. Usefulness of gene enrichment analyses depends on the quality of the database used [80]. If accurate, gene enrichment separates genes from those that were randomly identified during the RNAi screening and offers the largest source of antiviral target candidates for drug development, based on the assumption that all members of an enriched region are required for efficient viral replication. The fundamentals of network theory and its usage in GWAS analysis are reviewed in [80]. Gene expression studies can utilize enhanced methods of pathway analysis which go beyond treating pathways as simple sets of genes and incorporate the complex gene interactions described by the pathway, such as measurement of total pathway perturbation [81]. Unfortunately such methods require quantitative differential expression data to be applicable, a component lacking in these RNAi screening studies. It is possible, however, that inclusion of RNAi viral inhibition data in a modified version of this enhanced method might lead to improvement in pathway enrichment, but this is yet to be exemplified in the literature.

Thus, pathway analysis and gene function annotation offer the drug screening community automated procedures for extracting meaning from a large array of differentially expressed genes. The identified genes can be grouped according to the relationships among protein products, while potential drug targets associated with enriched pathways can be perceived.

**Figure 3:**
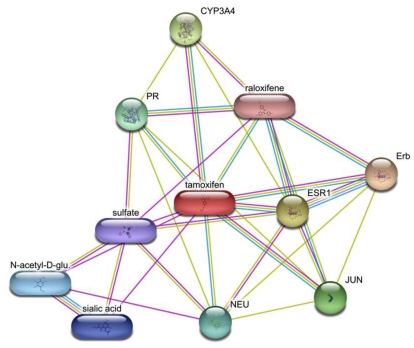
Gene interaction map overlapped with Tamoxifen via the STITCH database. The latter also connects ovals to one another suggesting that these molecules display similar biological behavior towards the same target. Edges refer to interactions as determined by experiment (purple), manual curation (cyan) or computationally predictions (yellow).

An illustration of the identification of therapeutic compounds by means of bioinformatics analysis is the use of a gene list to link Tamoxifen to the treatment of Systemic Lupus Erythematosus (SLE) [82–84]. Investigators discovered that the estrogen receptor pathway is a significantly enriched gene category with respect to a list of SLE’s aberrantly expressed genes through the Disease-Drug Correlation Ontology (DDCO). Other databases that similarly infuse chemical knowledge into the pathway databases include KEGG, the Connectivity Map, Ingenuity IPA and the STITCH database (all of which are also included in Table 1). Figure 3 offers an example of a gene interaction map annotated with small molecules.

While the previously cited resources offer investigators utmost convenience in immediately accessing lists of available small molecule modulators related to a pathway of interest, other databases connect small molecule modulators with known protein targets. These require a separate pathway analysis to choose a particular set of gene products of interest. Suitable resources include DrugBank [85,86], PDB/sc-PDB [87–89], PubChem [90], Sunset Molecular’s WOMBAT-PK 2010 [91,92] and the BRENDA database [93].

Following a bioinformatics selection of target candidates, individual targets must be selected for medicinal chemistry, for instance, based on the previous discovery of small-molecule blockers or the availability of crystal structures in the Protein Data Bank (PDB). [88,89]. Then, *de novo* drug discovery can be sustained through 3D virtual screening [94] and structure-based design. Application of these methods to the analysis of HIV and flu RNAi screens will be discussed in the next section.

## 3. Bioinformatics Approaches for Identifying Host-Factors Required for HIV Replication

Each of the systematic studies examined in the following sections employed a unique bioinformatics approach to pathway analysis. Similar to a chemoinformatics clustering analysis of a high-throughput screen to short-list a set of chemical leads for optimization, a goal of the RNAi screening studies is to identify, by means of gene pathway or functional analysis, potential host factor targets that are essential for viral replication. A key question is whether the resulting bioinformatics short list of host factors contains suitable candidates for drug development.

### 3.1. Bioinformatics Approaches to Identify Host-Factors Required for HIV Virus Replication

**Figure 4:**
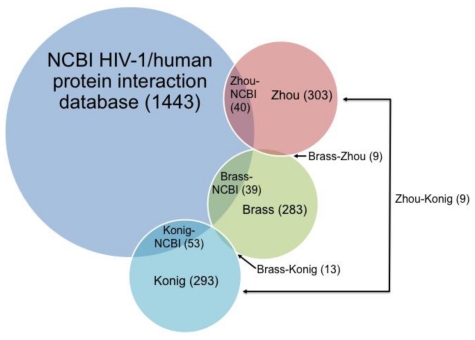
Illustration of the pairwise overlap between hit genes in the three HIV siRNA studies and the NCBI database. Circle areas are proportional to the number of genes. For clarity, three-way and higher overlaps are not shown.

For HIV, three independent siRNA studies were published in 2008 by Brass *et al.* [36], Konig *et al.* [95] and Zhou *et al.* [37]. All three siRNA studies utilized the National Center for Biotechnology Information (NCBI) database of HIV-1 and human protein interactions (currently 1443 proteins identified) to evaluate the overlap of hit genes with the curated virus-host interactions available in the NCBI database [61]. Figure 4 illustrates the total number of genes found as well as the pairwise overlap between genes in each study. A meta-analysis of these genome-wide studies was subsequently performed by Bushman *et al*. in 2009 [96].

Bushman *et al.* performed an overlap analysis/random distribution comparison based on these data and found associations that were statistically significant (*p*-values < 0.001). While one may safely assume that the hit genes are enriched with respect to independently identified and confirmed host factors required for HIV-1 replication, pairwise overlaps between the studies are low, ranging only from 3 to 6%. While these were still judged statistically significant (*p*-values < 0.024 for all pairs) [96], the overall very low redundancy suggests considerable experimental variability associated with each siRNA screen.

Variation in individual host factors could be accounted for by a number of factors, including: (a) high experimental variance of siRNA transfection efficiencies [42]; (b) harvest of cells at different time points post-infection; (c) the use of different analysis methods and filtering thresholds; (d) an inherent bias of individual assays towards specific stages of the viral life cycle [96]; and (e) overall moderate reproducibility of siRNA-based screens[97]. Some of these variances might be readily controlled by additional replicates examined per screen. For instance, only the study by Konig *et al.* performed the screen in duplicate. As a case in point, the experimental data showed large variances between the replicates: 24% of hit siRNAs (141) exhibit standard deviations greater than 25% of their median values. Furthermore, Bushman *et al.* demonstrated that adjusting the filtering thresholds in this study strongly influences the nature of the identified genes (shown Figure 1D of Bushman *et al.*) [96]. Other parameters, such as non-uniform harvesting time points, are inherent to the design of each individual study and cannot be standardized retroactively. Although capturing different stages of the viral life cycle in separate studies may ultimately be necessary to fully appreciate the scope of the host-pathogen interaction network, different analysis times should be considered as a major contributor to the low level of congruity between the currently available data.

Independent of redundancy between studies, the question remains of whether the gene hits represent *bona fide* host factors required for HIV replication or false positives that may have arisen from experimental variability. Equally important for hit confirmation is the organization of the data sets into groups by gene function and cellular pathways to illuminate distinct parts of the intricate host-pathogen interaction network. Using terms from the Gene Ontology (GO) database Brass *et al*. noted that 103 of their hit genes were assigned with 136 statistically significant (*p*-value < 0.05) biological processes [36]. In brief, the GO database is a consortium established to relate genes to one another in a fixed file format within three categories: biological processes, cellular components and molecular functions [44,63,98,99]. To reduce redundancy, these categories were clustered and manually curated. GO analysis yielded 17 enriched cellular functions in the Brass HIV study. Alternatively, the Zhou study used Ingenuity Pathway Analysis to determine enriched molecular functions and biological pathways. Thirty-two molecular functions were identified, and twelve biological processes were found to be statistically significant (*p*-value < 0.05).

**Figure 5:**
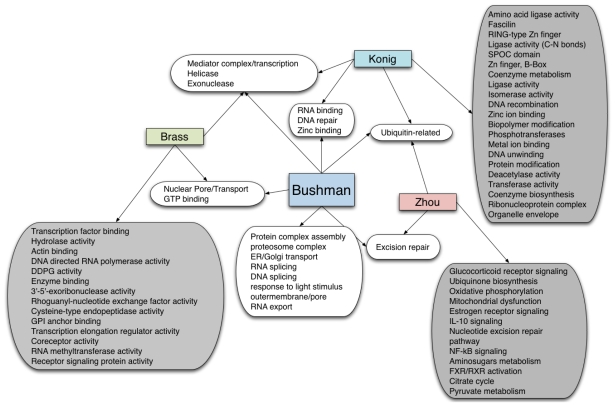
Comparison of HIV-dependent host functions identified by Bushman *et al*. [96], Brass *et al*. [36], Konig *et al*. [95] and Zhou *et al*. [37]. Grey boxes indicate functions unique to an individual study.

In contrast, Konig and colleagues employed a multi-tiered bioinformatics approach to identify the host factors most important to HIV replication through the use of the Prolexys HyNet database [95]. This resulted in networks of 2468, 4080, and 2850 genes in the HIV, MLV, AAV and toxicity assays, respectively. Using the Database for Annotation, Visualization, and Integrated Discovery (DAVID) [100], the Konig team extracted overrepresented functional clusters for all genes found in the three HIV siRNA studies. Filtering for significance (*p*-value < 0.06 based on a geometric mean for all the terms in a group), redundancy, biological relevance and specificity returned 24 functional groups (A listing of the overlapping pathways and those identified uniquely are presented in Figure 5), most of which contained genes that were identified in two or more studies. Although each study contains a significant number of genes that may be defined by molecular functions common to each of the three studies, consistent function identification across the siRNA screens is lacking due to the distinctions in each study’s bioinformatics methods. These functions may be defined differently between methods due to redundancies present in each database. Slightly different but biologically meaningless distinctions can arise thereby.

Although some well-documented host factors required for HIV replication (such as CD4, CXCR4, NFκB subunit RELA, activating kinases AKT1 and JAK1, TSG101, and various cofactors of Vpr, Vif, Tat, and Rev) [101–105] were identified in at least one of the three siRNA studies, a variety of other host factors known to engage with HIV (HLA-B57, HLA-C, PSIP1/LEDBF/p75, Sp1, cyclophilin A, ITGB1, ITGB2, and ITGB3) were not discovered [96,106–115]. Of these, the absence of the integration cofactor PSIP1/LEDBF/p75, HIV long terminal repeat transcription factor Sp1, HIV Gag binding protein cyclophilin A, and the three integrin proteins (ITBG1, ITBG2, ITBG3) is most noticeable. [108–112,114–117] These results suggest that even a combined host factor analysis is at risk of missing key host components required for viral replication. Furthermore, no single siRNA study thus far illuminates all relevant, and currently known, cellular factors associated with the pathogen.

The meta-analysis was extended by building an HIV-host factor interaction network of 1657 cellular proteins using an array of protein-protein interaction databases (BIND, HPRD, MINT, and Reactome) [96]. With MCODE’s graph theoretic clustering algorithm, clusters within this interactome map having different functions were identified. Of the 11 clusters found, 10 were associated with distinct cellular functions: the proteasome, transcription/RNA polymerase, the mediator complex, Tat activation/transcriptional elongation, RNA Binding/Splicing, BiP/GRP78/HSPA5 Chaperone, and CCT Chaperone. From a drug discovery perspective, however, small molecule testing or counter-screening with individual siRNAs against target candidates are required to validate individual pathways.

Lack of saturation in host genes identified through current siRNA screens, varying consistency and high overlap of genes in specific areas emerge as future challenges for application to system-wide drug discovery efforts.

### 3.2. Bioinformatics Approaches to Identify Host-Factors Required for Influenza Virus Replication

In addition to application of system-wide siRNA screens to the HIV system, the technology was applied to the influenza virus. Major siRNA studies were reported by Hao *et al*. [118], Brass *et al*. [119], Shapira *et al*. [120], Konig *et al*. [34] and Karlas *et al*. [35]. Other types of screens were performed by Josset *et al.* [121], which identified a gene list based on gene expression response to influenza; and Coombs *et al*. [122], which performed a quantitative analysis of protein level changes in infected cells. While Hao and colleagues employed a Drosophila cell-based host system for their siRNA screens, both Konig and Karlas relied on human lung cells (A549) and the influenza A/WSN (H1N1) strain or a recombinant variant thereof. Brass and colleagues used a human osteosarcoma cell system (U2OS) and the influenza A/PR/8 (H1N1) strain. The Shapira study is unique in that it combined results for yeast two- hybrid analyses, genome-wide transcriptional gene expression profiling and siRNA screening. Unfortunately, a single publically available resource similar to the NIH/NIAID HIV-1 interaction database does not exist for influenza virus, although many distinct virus-host interactions have been described in the literature (reviewed in [123]).

Watanabe *et al.* summarized five of the six systematic studies reported above and performed bioinformatics analysis on the 1,449 identified genes required for influenza replication [123]. Much like the Bushman *et al*. analysis of HIV host factors [96], 128 genes were found in multiple screens when pairwise comparisons were performed. The highest pairwise overlap (32 genes) is found in the Konig *et al*. and Karlas *et al*. studies, possibly due to the overlaps in host cell type (A549 cells) and influenza virus strain (A/WSN (H1N1)). This lack of overlap is also illustrated in the pairwise analysis given in Table S1. Unlike the meta-analysis for the HIV studies discussed above, this pairwise comparison lacked random distribution simulations, preventing the assessment of statistical significance. Nevertheless, the observed low overlap rate most likely results from factors similar to those discussed above for the HIV siRNA studies, *i.e.*, different harvest times, detection thresholds and host cell lines, coupled with the additional complication of variability introduced through the use of different viral strains.

As described in the HIV siRNA analyses, each study examining influenza virus infections performed individual bioinformatics analyses on siRNA screening results. A summary of these bioinformatics data along with the methodology is reviewed by Min [124]. In unique congruence, three of the influenza virus studies explored the use of known small-molecule inhibitors to obtain independent proof-of-concept for the importance of cellular targets identified by bioinformatics for virus replication [34,35,121].

Konig reported six compounds with EC_50_ values ranging from 0.5 to 30 μM target FRAP1, HSP90AA1, TUBB, FGFR4, GSK3B, or ANPEP. Of these, FRAP1, TUBB, FGFR4 and GSK3B home to the same GO Term cluster, protein kinase activity, recommending it as a potentially rich source for influenza virus inhibitors. The cytosolic chaperone Hsp90AAP1 was identified in a separate GO Term cluster; interestingly, previous reports have already established a link to influenza virus [125] and HCV [126] replication. The Karlas study reported another efficacious small molecule inhibitor, TG003, which targets the CDC-like kinase 1 (CLK1). CLK1 was retrieved from the Spliceosome GO term cluster, where it ranked seventh in significance among the list of enriched cellular components.

While the above studies identified potential host factor targets through GO term enrichment and then followed up with small molecules available for viral testing, the Josset project searched Connectivity Map with 20 of the most perturbed genes from the 300 initially identified. Of the eight compounds available through commercial vendors, six attenuated influenza virus replication with EC_50_ values ranging from 5.8 to 30 μM. In-house analysis of the 20 genes used to identify these compounds revealed that they are significantly enriched in metabolic processes. Similar to previous studies, the Josset report does not explicitly identify a host pathway essential for viral replication based on the small molecule inhibition studies. To appreciate the full potential of this approach for antiviral drug development, it may be informative to collect all known inhibitors of a particular host pathway and determine the complete extent of virus inhibition.

**Figure 6:**
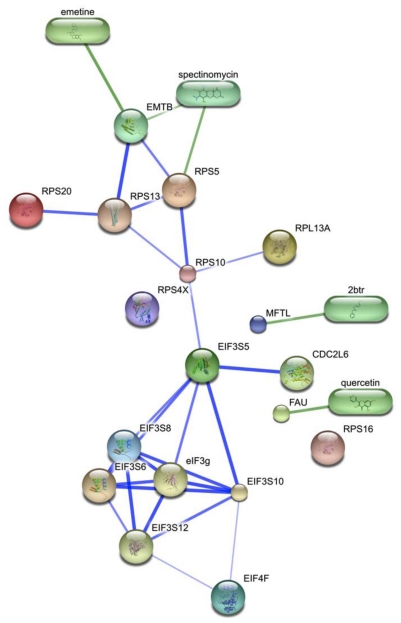
Small molecule (ovals) identification of gene products (spheres) associated with translation initiation. Green edges represent protein-ligand interactions. These compounds have not been reported previously to interfere with influenza infection, although quercetin has been demonstrated to attenuate HCV, however through a different host factor [126].

Watanabe *et al.* performed a meta-analysis of the siRNA results using the set of 128 genes found in two or more studies [123]. The major gene categories were determined through PANTHER, a database that also utilizes GO terms to organize gene lists. Several molecular functions were found significant: nucleic acid-binding proteins, kinases, transcription factors, ribosomal proteins, hydrogen transporters and proteins related to mRNA splicing. Biological processes found to be consequential were protein metabolism and modification, signal transduction, protein phosphorylation, nucleoside, nucleotide and nucleic acid metabolism and intracellular transport. Reactome analysis tagged as significant eukaryotic translation initiation, regulation of gene expression, processing of capped intron-containing pre-mRNAs and Golgi-to-ER retrograde transport. This set of 128 genes was further integrated with the viral protein interaction partners determined by Konig and Shapira, resulting in a network of virus-host interactions. Based on this map, MCODE further identified translation initiation, mRNA processing and proton-transport as crucial. Accordingly, mining of the top MCODE cluster in Figure 6 predicts that compounds such as spectoinomycin, emetine and quercetin will interfere with influenza virus replication.

Successful outcomes for bioinformatics searches predominantly depend on the accuracy of tabulated database interactions. As detailed below, use of different databases may alter the profile of pathways that are enriched from the same gene list. In such cases, users are obligated to formulate a realistic biological interpretation of the relational data to ensure identification of meaningful candidate compounds for an antiviral drug program.

## 4. Pathway Database Comparisons: Same Source, Different Interpretation

As outlined above, it is a primary function of gene databases to extract biological meaning as well as potential therapeutic host factors from a high throughput RNAi screen by means of descriptive annotations of genes common to a particular biological pathway or gene function. In the realm of antiviral drug discovery, this approach aims at identifying host cell components critical for virus replication.

Crucial for the success of this strategy is the quality of the pathway database used, which is determined by the curation method of published experimental data of gene associations and the expertise of the curators involved. Soh *et al*. have demonstrated that inconsistencies emerge when gene association data are compared across different pathways databases [127]. This came as a surprise, since most databases share published literature as a data source, suggesting that methodology for curation and criteria for gene associations were not uniform (for this study, the Ingenuity IPA, KEGG, and Wikipathways databases were compared). Assuming curation is performed on available literature data, however, one expects similar genes and gene pairs to be found across the different databases. (Gene pairings are defined as gene product associations confirmed by the database curator).

The Wnt signaling pathway provides a tangible example illustrating the current challenges. This pathway has been implicated in therapeutic interference with cancer and viral entry. Two-way analyses revealed approximately 80% gene similarity when based on the KEGG and Wikipathways databases (Table 4 in [127]). However, only 43% similarity is found when Ingenuity and KEGG are examined (Table 3 in [127]), while comparison of Ingenuity and Wikipathways databases returns only 28% similarity (Table 5 in [127]). Inconsistencies across databases are even more disconcerting when gene pair overlaps are examined: KEGG/Wikipathways (18%), Ingenuity/KEGG (8%), Ingenuity/Wikipathways (0%). It is the quality of the gene pairing data in each database, however, that allows end users to triage the multiple RNAi screening results for pathway congruity.

Looking on a broader scale across 26 cellular pathways described in Soh *et al*., gene overlap similarity has a mean value of 66.5% when comparing the KEGG database and Wikipathways [127]. By contrast, the mean values of similarity for Ingenuity/KEGG and Ingenuity/Wikipathways were 53.8% (12 pathway categories) and 41% (11 pathway categories), respectively. Despite the higher gene overlap between KEGG and Wikipathways, the pairing overlap is still only approximately 50% for any listed pathway compared across any three of the databases. KEGG is curated by a single lab group, while Wikipathways is curated through a community effort. At the moment, it is not clear to what extent the curation procedures contribute to the highly variable data mismatches. However, there is little doubt that this and other variables would benefit from cross-consolidation between the various databases.

Soh *et al.* also analyzed the comprehensiveness of the databases, which was a measure for the total number of genes from all three databases [127]. This was followed by evaluation of gene members and pairings of each database against the pool, which consisted of 21,314 genes and 60,900 pairings. KEGG was shown to be the most comprehensive of the three databases, but this was influenced by the result of KEGG’s inclusion of metabolic pathways specifically not curated by either of the other databases.

Concentrating in particular on viral host factors, we performed an in-house analysis that compares host proteins involved in the influenza virus life cycle across various databases. Databases used in this example included Reactome, Ingenuity IPA and PANTHER. The Reactome database records six host factor genes for influenza in each the categories associated with NS1-mediated effects and virus-induced apoptosis. Databases such as Ingenuity IPA and PANTHER lack pathway categories dedicated to influenza virus. Keyword searches for influenza in the PANTHER database identified no host factor associated with influenza virus infection [58]. Conversely, keyword searching of the Ingenuity database generated a list of five signaling pathways (Lipids/Lipid Rafts, MAPK, PI3K/AKT, Wnt/GSK-3β, hypercytokinemia) involved in the pathogenesis of influenza virus, constituting a list of 38 genes [128]. Import of the latter into all other databases allows the genes to be categorized into signaling pathways such as Wnt, PI3K, and MAPK. However, database annotations suggest that Ingenuity is more likely to alert the user to the genes’ roles in influenza virus infection.

Applying this approach to the previously described influenza RNAi screens, we sought to address the question of how does target identification change when different pathway databases are applied to the same dataset? Databases used in this comparison were PANTHER, Reactome and STRING, and the data set analyzed was the commonly identified 128 gene list generated by Watanabe *et al.* [123]. Results are presented in Table 2 with reference to the Wanatabe analysis of the same genes.

It becomes immediately obvious when examining the most enriched pathways that only the STRING database seems to reproduce the results generated by Watanabe *et al*. using GeneGO/MCODE. In all other cases, the different databases returned remarkably different top pathways when the same gene expression set was analyzed. Closer examination reveals that that other top ranking pathways (*i.e.*, translation initiation) rank lower on the Reactome enrichment analysis scale. Pathways associated with B-Cell metabolism are also identified by the Ingenuity IPA and Reactome enrichments, although slightly different naming schemes are used. Since discrete databases identify certain similar pathways at different rankings, a consensus scoring function applicable to available databases appears warranted. This would afford greater confidence in the identification of individual targets for follow-up through small molecule searching.

## 5. Conclusions

Previous GWAS experiments have attempted to capture the most relevant cellular host pathways utilized by pathogens such as HIV and influenza virus for virus replication [96,123]. As shown by reviewers such as Bushman *et al.* and Watanabe *et al.*, gene lists and enriched pathways vary widely despite the pursuit of similar biological goals. Indeed, the likelihood to successfully identify novel host-directed antivirals would increase significantly if the reproducibility of individual RNAi screens were to be increased [97]. Further challenges emerge from differently curated pathway databases that return unrelated enriched pathways based on analysis of the same gene data set. Preliminary analysis of this situation using the Watanabe 128 pairwise genes suggests that a consensus scoring protocol applicable across different databases would be desirable to clarify this issue. Despite these hurdles associated with experimental false positives and the complexities inherent in interpreting pairwise gene interactions, several tangible examples (*i.e.*, Konig *et al.*, Karlas *et al*., and Josset *et al.*) demonstrate that RNAi screening coupled with bioinformatics-driven triaging is a viable method to identify small molecule inhibitors of virus replication.

Current databases that infuse chemical knowledge into schemes such as Ingenuity IPA and the Connectivity Map are limited to a small number of compounds, mostly FDA-approved drugs. This narrow focus limits their application to current translational medicine. The STITCH database makes an interesting leap by crosslinking its gene network with multiple chemical-genomic high throughput screening results archived in PubChem. These experimental chemicals along with compounds currently tested *in vitro* for various endpoints offer a rich source for hit candidates with optimization potential. As more databases are used to analyze potential host targets, validation methods employing siRNA are improved and small molecule knowledge is added to the genetic web, more drug discovery initiatives are likely to incorporate this approach in their portfolio of standard operations for the identification of antiviral therapeutics.

## Supporting Information

**Table S1:** Pairwise comparison of influenza genome-wide studies.

	Konig [129]	Karlas [130]	Brass [131]	Shapira [132]	Josset [133]	Coombs [134]
Konig		32	9	16	10	1
Karlas	32		12	18	5	3
Brass	9	12		10	6	2
Shapira	16	18	10		20	15
Josset	10	5	6	20		3
Coombs	1	3	2	15	3

## Figures and Tables

**Table 1:** Commercial and open source pathway databases.

Database	Description	References
Kyoto Encyclopedia of Genes and Genome (KEGG)	Public Resource links genes to crystal structures and drugs when information is available	[52–54]
Reactome	Public Resource accepts a gene list for the pathway analyzer and returns percentage population per pathway	[55–57]
Protein Analysis Through Evolutionary Relationships (PANTHER)	Free pathway database allows user to identify enrichment in biological pathways, GO terms or protein class	[58,59]
WikiPathways	Community curated pathway database	[60,61]
Ingenuity IPA	Commercial pathway database to identify enrichment in pathways/GO terms; links drugs to specific genes	[62]
Gene Ontology (GO) Consortium	Community database that clusters genes by biological process, molecular function or cellular location across multiple species	[63]
Search Tool for the Retrieval of Interacting Genes/Proteins (STRING)	Freely available functional relationships database displays direct neighborhood relationships between proteins that interact directly or through an intermediary	[64–69]
Search Tool for Interactions of Chemicals (STITCH)	Crosslinks gene products with chemical structures from PubChem	[70,71]
GeneGo Metacore	Commercial manually curated pathway database annotated with 600,000 compounds	[72]
Prolexys HyNet	Commercial database protein-protein interaction identified via in-house yeast two-hybrid screening	[73]
Biomolecular Interaction Network Database (BIND)	Free and Commercial versions describing protein-protein interactions, molecular complexes and pathways	[74,75]
Molecular Interactions Database (MINT)	Public protein-protein interaction database based on peerreviewed literature. Accessible through web-interface or Simple Object Access Protocol/Representational State Transfer (SOAP/REST) protocols	[76]
Human Protein Reference Database (HPRD)	Public proteonomic database with descriptions for 2750 human proteins taken from the primary literature	[77–79]

**Table 2:** Comparison of pathway results from the Watanabe pairwise influenza gene set.

GeneGO/MCODE	STRING	PANTHER	Ingenuity IPA	Reactome
Translation Initiation	Translation Initiation	Apoptosis signaling pathway	Chronic Myeloid Leukemia Signaling	Dissolution of Fibrin Clot
Pre-mRNA Processing	Pre-mRNA Processing	T cell activation	B Cell Receptor Signaling	Influenza Life Cycle
Proton-Transporter V-type ATPase	Proton-Transporter V-type ATPase	Angiogenesis	Production of Nitric Oxide and Reactive Oxygen Species in Macrophages	MAP kinase cascade
	COPI coating of Golgi vesicle	Toll receptor signaling	EIF2 Signaling	Metabolism of nitric oxide
	Nuclear Transport	Inflammation mediated by chemokine and cytokine signaling pathways	Rank Signaling in Osteoclast	Eukaryotic Translation Initiation
		Cell cycle	CD40 Signaling	Signaling by FGFR
		PDGF signaling	Molecular Mechanisms of Cancer	Eukaryotic Translation Termination
		FGF signaling	Role of PKR in Interferon Induction and Antiviral Response	Eukaryotic Translation Elongation
		FAS signaling		Regulation of beta-cell development
		Ras Pathway		Signaling by Insulin receptor
		B cell activation		Processing of Capped Intron-Containing Pre-mRNA

## References

[1] Coen DM, Richman DD, Knipe DM, Howley PM (2007). Antiviral Agents. Fields Virology.

[2] Moore C, Galiano M, Lackenby A, Abdelrahman T, Barnes R, Evans MR, Fegan C, Froude S, Hastings M, Knapper S (2011). Evidence of person-to-person transmission of oseltamivir-resistant pandemic influenza A(H1N1) 2009 virus in a hematology unit. J. Infect. Dis.

[3] Sheu TG, Fry AM, Garten RJ, Deyde VM, Shwe T, Bullion L, Peebles PJ, Li Y, Klimov AI, Gubareva LV (2011). Dual resistance to adamantanes and oseltamivir among seasonal influenza A(H1N1) viruses: 2008–2010. J. Infect. Dis.

[4] Hayden FG, de Jong MD (2011). Emerging influenza antiviral resistance threats. J. Infect. Dis.

[5] Walsh C (2000). Molecular mechanisms that confer antibacterial drug resistance. Nature.

[6] Cegelski L, Marshall GR, Eldridge GR, Hultgren SJ (2008). The biology and future prospects of antivirulence therapies. Nat. Rev. Microbiol.

[7] Schinazi RF, Peters J, Williams CC, Chance D, Nahmias AJ (1982). Effect of combinations of acyclovir with vidarabine or its 5′-monophosphate on herpes simplex viruses in cell culture and in mice. Antimicrob. Agents Chemother.

[8] Kellam P (2006). Attacking pathogens through their hosts. Genome Biol.

[9] Schwegmann A, Brombacher F (2008). Host-directed drug targeting of factors hijacked by pathogens. Sci Signal.

[10] Tan SL, Ganji G, Paeper B, Proll S, Katze MG (2007). Systems biology and the host response to viral infection. Nat. Biotechnol.

[11] Koon HB, Bubley GJ, Pantanowitz L, Masiello D, Smith B, Crosby K, Proper J, Weeden W, Miller TE, Chatis P (2005). Imatinib-induced regression of AIDS-related Kaposi’s sarcoma. J. Clin. Oncol.

[12] Salerno D, Hasham MG, Marshall R, Garriga J, Tsygankov AY, Grana X (2007). Direct inhibition of CDK9 blocks HIV-1 replication without preventing T-cell activation in primary human peripheral blood lymphocytes. Gene.

[13] Reeves PM, Bommarius B, Lebeis S, McNulty S, Christensen J, Swimm A, Chahroudi A, Chavan R, Feinberg MB, Veach D (2005). Disabling poxvirus pathogenesis by inhibition of Abl-family tyrosine kinases. Nat. Med.

[14] Hirsch AJ, Medigeshi GR, Meyers HL, DeFilippis V, Fruh K, Briese T, Lipkin WI, Nelson JA (2005). The Src family kinase c-Yes is required for maturation of West Nile virus particles. J. Virol.

[15] Pearson G, Robinson F, Beers Gibson T, Xu BE, Karandikar M, Berman K, Cobb MH (2001). Mitogen-activated protein (MAP) kinase pathways: Regulation and physiological functions. Endocr. Rev.

[16] Pleschka S, Wolff T, Ehrhardt C, Hobom G, Planz O, Rapp UR, Ludwig S (2001). Influenza virus propagation is impaired by inhibition of the Raf/MEK/ERK signalling cascade. Nat. Cell Biol.

[17] Zhu H, Cong JP, Yu D, Bresnahan WA, Shenk TE (2002). Inhibition of cyclooxygenase 2 blocks human cytomegalovirus replication. Proc. Natl. Acad. Sci. USA.

[18] Goldman JM, Druker BJ (2001). Chronic myeloid leukemia: current treatment options. Blood.

[19] Ludwig S (2009). Targeting cell signalling pathways to fight the flu: Towards a paradigm change in anti-influenza therapy. J. Antimicrob. Chemother.

[20] Newsome TP, Scaplehorn N, Way M (2004). SRC mediates a switch from microtubule- to actin-based motility of vaccinia virus. Science.

[21] Kujime K, Hashimoto S, Gon Y, Shimizu K, Horie T (2000). p38 mitogen-activated protein kinase and c-jun-NH2-terminal kinase regulate RANTES production by influenza virus-infected human bronchial epithelial cells. J. Immunol.

[22] Yoon JJ, Chawla D, Paal T, Ndungu M, Du Y, Kurtkaya S, Sun A, Snyder JP, Plemper RK (2008). High-throughput screening-based identification of paramyxovirus inhibitors. J. Biomol. Screen.

[23] Bushell KM, Sollner C, Schuster-Boeckler B, Bateman A, Wright GJ (2008). Large-scale screening for novel low-affinity extracellular protein interactions. Genome Res.

[24] Fields S, Sternglanz R (1994). The two-hybrid system: an assay for protein-protein interactions. Trends Genet.

[25] Chien CT, Bartel PL, Sternglanz R, Fields S (1991). The two-hybrid system: A method to identify and clone genes for proteins that interact with a protein of interest. Proc. Natl. Acad. Sci. USA.

[26] Kaur G, Roy I (2008). Therapeutic applications of aptamers. Expert Opin. Invest. Drugs.

[27] Borghouts C, Kunz C, Groner B (2008). Peptide aptamer libraries. Comb. Chem. High Throughput Screen.

[28] Grimm D, Kay MA (2007). Therapeutic application of RNAi: Is mRNA targeting finally ready for prime time?. J. Clin. Invest.

[29] Fewell GD, Schmitt K (2006). Vector-based RNAi approaches for stable, inducible and genome-wide screens. Drug Discov. Today.

[30] Filipowicz W, Bhattacharyya SN, Sonenberg N (2008). Mechanisms of post-transcriptional regulation by microRNAs: Are the answers in sight?. Nat. Rev. Genet.

[31] Stenvang J, Kauppinen S (2008). MicroRNAs as targets for antisense-based therapeutics. Expert Opin. Biol. Ther.

[32] Nguyen DG, Yin H, Zhou Y, Wolff KC, Kuhen KL, Caldwell JS (2007). Identification of novel therapeutic targets for HIV infection through functional genomic cDNA screening. Virology.

[33] Basha S, Rai P, Poon V, Saraph A, Gujraty K, Go MY, Sadacharan S, Frost M, Mogridge J, Kane RS (2006). Polyvalent inhibitors of anthrax toxin that target host receptors. Proc. Natl. Acad. Sci. USA.

[34] Konig R, Stertz S, Zhou Y, Inoue A, Hoffmann HH, Bhattacharyya S, Alamares JG, Tscherne DM, Ortigoza MB, Liang Y (2010). Human host factors required for influenza virus replication. Nature.

[35] Karlas A, Machuy N, Shin Y, Pleissner KP, Artarini A, Heuer D, Becker D, Khalil H, Ogilvie LA, Hess S (2010). Genome-wide RNAi screen identifies human host factors crucial for influenza virus replication. Nature.

[36] Brass AL, Dykxhoorn DM, Benita Y, Yan N, Engelman A, Xavier RJ, Lieberman J, Elledge SJ (2008). Identification of host proteins required for HIV infection through a functional genomic screen. Science.

[37] Zhou H, Xu M, Huang Q, Gates AT, Zhang XD, Castle JC, Stec E, Ferrer M, Strulovici B, Hazuda DJ, Espeseth AS (2008). Genome-scale RNAi screen for host factors required for HIV replication. Cell Host Microbe.

[38] Borner K, Hermle J, Sommer C, Brown NP, Knapp B, Glass B, Kunkel J, Torralba G, Reymann J, Beil N (2010). From experimental setup to bioinformatics: An RNAi screening platform to identify host factors involved in HIV-1 replication. Biotechnol. J.

[39] Pache L, Konig R, Chanda SK (2011). Identifying HIV-1 host cell factors by genome-scale RNAi screening. Methods.

[40] Krishnan MN, Ng A, Sukumaran B, Gilfoy FD, Uchil PD, Sultana H, Brass AL, Adametz R, Tsui M, Qian F (2008). RNA interference screen for human genes associated with West Nile virus infection. Nature.

[41] Li Q, Brass AL, Ng A, Hu Z, Xavier RJ, Liang TJ, Elledge SJ (2009). A genome-wide genetic screen for host factors required for hepatitis C virus propagation. Proc. Natl. Acad. Sci. USA.

[42] Sigoillot FD, King RW (2011). Vigilance and Validation: Keys to Success in RNAi Screening. ACS Chem. Biol.

[43] Huang DW, Sherman BT, Lempicki RA (2009). Bioinformatics enrichment tools: paths toward the comprehensive functional analysis of large gene lists. Nucleic Acids Res.

[44] Bard JB, Rhee SY (2004). Ontologies in biology: design, applications and future challenges. Nat. Rev. Genet.

[45] Rhee SY, Wood V, Dolinski K, Draghici S (2008). Use and misuse of the gene ontology annotations. Nat. Rev. Genet.

[46] Chuang HY, Hofree M, Ideker T (2010). A decade of systems biology. Annu. Rev. Cell Dev. Biol.

[47] Jensen LJ, Bork P (2010). Ontologies in quantitative biology: a basis for comparison, integration, and discovery. PLoS Biol.

[48] Khatri P, Draghici S (2005). Ontological analysis of gene expression data: Current tools, limitations, and open problems. Bioinformatics.

[49] Ooi HS, Schneider G, Chan YL, Lim TT, Eisenhaber B, Eisenhaber F (2010). Databases of protein-protein interactions and complexes. Methods Mol. Biol.

[50] Ooi HS, Schneider G, Lim TT, Chan YL, Eisenhaber B, Eisenhaber F (2010). Biomolecular pathway databases. Methods Mol. Biol.

[51] Quackenbush J (2001). Computational analysis of microarray data. Nat. Rev. Genet.

[52] Kanehisa M, Goto S, Furumichi M, Tanabe M, Hirakawa M (2010). KEGG for representation and analysis of molecular networks involving diseases and drugs. Nucleic Acids Res.

[53] Kanehisa M, Goto S, Hattori M, Aoki-Kinoshita KF, Itoh M, Kawashima S, Katayama T, Araki M, Hirakawa M (2006). From genomics to chemical genomics: new developments in KEGG. Nucleic Acids Res.

[54] Kanehisa M, Goto S (2000). KEGG: Kyoto encyclopedia of genes and genomes. Nucleic Acids Res.

[55] Matthews L, Gopinath G, Gillespie M, Caudy M, Croft D, de Bono B, Garapati P, Hemish J, Hermjakob H, Jassal B (2009). Reactome knowledgebase of human biological pathways and processes. Nucleic Acids Res.

[56] Vastrik I, D’Eustachio P, Schmidt E, Gopinath G, Croft D, de Bono B, Gillespie M, Jassal B, Lewis S, Matthews L (2007). Reactome: A knowledge base of biologic pathways and processes. Genome Biol.

[57] Joshi-Tope G, Vastrik I, Gopinath GR, Matthews L, Schmidt E, Gillespie M, D’Eustachio P, Jassal B, Lewis S, Wu G (2003). The Genome Knowledgebase: A resource for biologists and bioinformaticists. Cold Spring Harb. Symp. Quant. Biol.

[58] Thomas PD, Campbell MJ, Kejariwal A, Mi H, Karlak B, Daverman R, Diemer K, Muruganujan A, Narechania A (2003). PANTHER: A library of protein families and subfamilies indexed by function. Genome Res.

[59] Thomas PD, Kejariwal A, Campbell MJ, Mi H, Diemer K, Guo N, Ladunga I, Ulitsky-Lazareva B, Muruganujan A, Rabkin S (2003). PANTHER: A browsable database of gene products organized by biological function, using curated protein family and subfamily classification. Nucleic Acids Res.

[60] Pico AR, Kelder T, van Iersel MP, Hanspers K, Conklin BR, Evelo C (2008). WikiPathways: Pathway editing for the people. PLoS Biol.

[61] Karp PD, Ouzounis CA, Moore-Kochlacs C, Goldovsky L, Kaipa P, Ahren D, Tsoka S, Darzentas N, Kunin V, Lopez-Bigas N (2005). Expansion of the BioCyc collection of pathway/genome databases to 160 genomes. Nucleic Acids Res.

[62] Lee SJ, Ways JA, Barbato JC, Essig D, Pettee K, DeRaedt SJ, Yang S, Weaver DA, Koch LG, Cicila GT (2005). Gene expression profiling of the left ventricles in a rat model of intrinsic aerobic running capacity. Physiol. Genomics.

[63] Gene Ontology (GO) http://www.geneontology.org/.

[64] Szklarczyk D, Franceschini A, Kuhn M, Simonovic M, Roth A, Minguez P, Doerks T, Stark M, Muller J, Bork P (2011). The STRING database in 2011: Functional interaction networks of proteins, globally integrated and scored. Nucleic Acids Res.

[65] Jensen LJ, Kuhn M, Stark M, Chaffron S, Creevey C, Muller J, Doerks T, Julien P, Roth A, Simonovic M (2009). STRING 8—A global view on proteins and their functional interactions in 630 organisms. Nucleic Acids Res.

[66] von Mering C, Jensen LJ, Kuhn M, Chaffron S, Doerks T, Kruger B, Snel B, Bork P (2007). STRING 7—recent developments in the integration and prediction of protein interactions. Nucleic Acids Res.

[67] von Mering C, Jensen LJ, Snel B, Hooper SD, Krupp M, Foglierini M, Jouffre N, Huynen MA, Bork P (2005). STRING: Known and predicted protein-protein associations, integrated and transferred across organisms. Nucleic Acids Res.

[68] von Mering C, Huynen M, Jaeggi D, Schmidt S, Bork P, Snel B (2003). STRING: A database of predicted functional associations between proteins. Nucleic Acids Res.

[69] Snel B, Lehmann G, Bork P, Huynen MA (2000). STRING: A web-server to retrieve and display the repeatedly occurring neighbourhood of a gene. Nucleic Acids Res.

[70] Kuhn M, Szklarczyk D, Franceschini A, Campillos M, von Mering C, Jensen LJ, Beyer A, Bork P (2010). STITCH 2: An interaction network database for small molecules and proteins. Nucleic Acids Res.

[71] Kuhn M, von Mering C, Campillos M, Jensen LJ, Bork P (2008). STITCH: Interaction networks of chemicals and proteins. Nucleic Acids Res.

[72] Dezso Z, Nikolsky Y, Nikolskaya T, Miller J, Cherba D, Webb C, Bugrim A (2009). Identifying disease-specific genes based on their topological significance in protein networks. BMC Syst Biol.

[73] LaCount DJ, Vignali M, Chettier R, Phansalkar A, Bell R, Hesselberth JR, Schoenfeld LW, Ota I, Sahasrabudhe S, Kurschner C (2005). A protein interaction network of the malaria parasite Plasmodium falciparum. Nature.

[74] Bader GD, Hogue CW (2000). BIND—a data specification for storing and describing biomolecular interactions, molecular complexes and pathways. Bioinformatics.

[75] Bader GD, Donaldson I, Wolting C, Ouellette BF, Pawson T, Hogue CW (2001). BIND—The Biomolecular Interaction Network Database. Nucleic Acids Res.

[76] Ceol A, Chatr Aryamontri A, Licata L, Peluso D, Briganti L, Perfetto L, Castagnoli L, Cesareni G (2010). MINT, the molecular interaction database: 2009 update. Nucleic Acids Res.

[77] Prasad TS, Kandasamy K, Pandey A (2009). Human protein reference database and human proteinpedia as discovery tools for systems biology. Methods Mol. Biol.

[78] Mishra GR, Suresh M, Kumaran K, Kannabiran N, Suresh S, Bala P, Shivakumar K, Anuradha N, Reddy R, Raghavan TM (2006). Human protein reference database—2006 update. Nucleic Acids Res.

[79] Peri S, Navarro JD, Amanchy R, Kristiansen TZ, Jonnalagadda CK, Surendranath V, Niranjan V, Muthusamy B, Gandhi TK, Gronborg M (2003). Development of human protein reference database as an initial platform for approaching systems biology in humans. Genome Res.

[80] Diez D, Wheelock AM, Goto S, Haeggstrom JZ, Paulsson-Berne G, Hansson GK, Hedin U, Gabrielsen A, Wheelock CE (2010). The use of network analyses for elucidating mechanisms in cardiovascular disease. Mol. Biosyst.

[81] Tarca AL, Draghici S, Khatri P, Hassan SS, Mittal P, Kim JS, Kim CJ, Kusanovic JP, Romero R (2009). A novel signaling pathway impact analysis. Bioinformatics.

[82] Qu XA, Gudivada RC, Jegga AG, Neumann EK, Aronow BJ (2009). Inferring novel disease indications for known drugs by semantically linking drug action and disease mechanism relationships. BMC Bioinformatics.

[83] Sthoeger ZM, Zinger H, Mozes E (2003). Beneficial effects of the anti-oestrogen tamoxifen on systemic lupus erythematosus of (NZBxNZW)F1 female mice are associated with specific reduction of IgG3 autoantibodies. Ann. Rheum. Dis.

[84] Cohen-Solal JF, Jeganathan V, Grimaldi CM, Peeva E, Diamond B (2006). Sex hormones and SLE: influencing the fate of autoreactive B cells. Curr. Top. Microbiol. Immunol.

[85] Wishart DS, Knox C, Guo AC, Cheng D, Shrivastava S, Tzur D, Gautam B, Hassanali M (2008). DrugBank: A knowledgebase for drugs, drug actions and drug targets. Nucleic Acids Res.

[86] Wishart DS, Knox C, Guo AC, Shrivastava S, Hassanali M, Stothard P, Chang Z, Woolsey J (2006). DrugBank: A comprehensive resource for in silico drug discovery and exploration. Nucleic Acids Res.

[87] Kellenberger E, Muller P, Schalon C, Bret G, Foata N, Rognan D (2006). sc-PDB: An annotated database of druggable binding sites from the Protein Data Bank. J. Chem. Inf. Model.

[88] Berman HM, Bhat TN, Bourne PE, Feng Z, Gilliland G, Weissig H, Westbrook J (2000). The Protein Data Bank and the challenge of structural genomics. Nat. Struct. Biol.

[89] Berman HM, Westbrook J, Feng Z, Gilliland G, Bhat TN, Weissig H, Shindyalov IN, Bourne PE (2000). The Protein Data Bank. Nucleic Acids Res.

[90] Li Q, Cheng T, Wang Y, Bryant SH (2010). PubChem as a public resource for drug discovery. Drug Discov. Today.

[91] Olah M, Rad R, Ostopovici L, Bora A, Hadaruga N, Hadaruga R, Moldovan R, Fulias A, Mracec M, Opera TI, Schreiber SL, Kapoor TM, Wess G (2007). WOMBAT and WOMBAT-PK: Bioactivity Databases for Lead and Drug Discovery. Chemical Biology: From Small Molecules to Systems Biology and Drug Design.

[92] Opera TI, Benedetti P, Berellini G, Olah M, Fejgin K, Boyer S, Cruciani G (2006). Rapid ADME Filters for Lead Discovery. Molecular Interactions Field.

[93] Scheer M, Grote A, Chang A, Schomburg I, Munaretto C, Rother M, Sohngen C, Stelzer M, Thiele J, Schomburg D (2011). BRENDA, the enzyme information system in 2011. Nucleic Acids Res.

[94] Shoichet BK (2004). Virtual screening of chemical libraries. Nature.

[95] Konig R, Zhou YY, Elleder D, Diamond TL, Bonamy GMC, Irelan JT, Chiang CY, Tu BP, De Jesus PD, Lilley CE (2008). Global analysis of host-pathogen interactions that regulate early-stage HIV-1 replication. Cell.

[96] Bushman FD, Malani N, Fernandes J, D’Orso I, Cagney G, Diamond TL, Zhou HL, Hazuda DJ, Espeseth AS, Konig R (2009). Host Cell Factors in HIV Replication: Meta-Analysis of Genome-Wide Studies. PLoS Pathog.

[97] Barrows NJ, Le Sommer C, Garcia-Blanco MA, Pearson JL (2010). Factors affecting reproducibility between genome-scale siRNA-based screens. J. Biomol. Screen.

[98] Gene Ontology Consortium (2010). The Gene Ontology in 2010: Extensions and refinements. Nucleic Acids Res.

[99] The Reference Genome Group of the Gene Ontology Consortium (2009). The Gene Ontology’s Reference Genome Project: A unified framework for functional annotation across species. PLoS Comput. Biol.

[100] Dennis G, Sherman BT, Hosack DA, Yang J, Gao W, Lane HC, Lempicki RA (2003). DAVID: Database for Annotation, Visualization, and Integrated Discovery. Genome Biol.

[101] Fu W, Sanders-Beer BE, Katz KS, Maglott DR, Pruitt KD, Ptak RG (2009). Human immunodeficiency virus type 1, human protein interaction database at NCBI. Nucleic Acids Res.

[102] Garrus JE, von Schwedler UK, Pornillos OW, Morham SG, Zavitz KH, Wang HE, Wettstein DA, Stray KM, Cote M, Rich RL (2001). Tsg101 and the vacuolar protein sorting pathway are essential for HIV-1 budding. Cell.

[103] Kilzer JM, Stracker T, Beitzel B, Meek K, Weitzman M, Bushman FD (2003). Roles of host cell factors in circularization of retroviral dna. Virology.

[104] Wei P, Garber ME, Fang SM, Fischer WH, Jones KA (1998). A novel CDK9-associated C-type cyclin interacts directly with HIV-1 Tat and mediates its high-affinity, loop-specific binding to TAR RNA. Cell.

[105] Yedavalli VS, Neuveut C, Chi YH, Kleiman L, Jeang KT (2004). Requirement of DDX3 DEAD box RNA helicase for HIV-1 Rev-RRE export function. Cell.

[106] Fellay J, Shianna KV, Ge D, Colombo S, Ledergerber B, Weale M, Zhang K, Gumbs C, Castagna A, Cossarizza A (2007). A whole-genome association study of major determinants for host control of HIV-1. Science.

[107] Matthews PC, Prendergast A, Leslie A, Crawford H, Payne R, Rousseau C, Rolland M, Honeyborne I, Carlson J, Kadie C (2008). Central role of reverting mutations in HLA associations with human immunodeficiency virus set point. J. Virol.

[108] Cherepanov P (2007). LEDGF/p75 interacts with divergent lentiviral integrases and modulates their enzymatic activity in vitro. Nucleic Acids Res.

[109] Cherepanov P, Maertens G, Proost P, Devreese B, Van Beeumen J, Engelborghs Y, De Clercq E, Debyser Z (2003). HIV-1 integrase forms stable tetramers and associates with LEDGF/p75 protein in human cells. J. Biol. Chem.

[110] Ciuffi A, Bushman FD (2006). Retroviral DNA integration: HIV and the role of LEDGF/p75. Trends Genet.

[111] Gamble TR, Vajdos FF, Yoo S, Worthylake DK, Houseweart M, Sundquist WI, Hill CP (1996). Crystal structure of human cyclophilin A bound to the amino-terminal domain of HIV-1 capsid. Cell.

[112] Llano M, Saenz DT, Meehan A, Wongthida P, Peretz M, Walker WH, Teo W, Poeschla EM (2006). An essential role for LEDGF/p75 in HIV integration. Science.

[113] Maertens G, Cherepanov P, Pluymers W, Busschots K, De Clercq E, Debyser Z, Engelborghs Y (2003). LEDGF/p75 is essential for nuclear and chromosomal targeting of HIV-1 integrase in human cells. J. Biol. Chem.

[114] Marshall HM, Ronen K, Berry C, Llano M, Sutherland H, Saenz D, Bickmore W, Poeschla E, Bushman FD (2007). Role of PSIP1/LEDGF/p75 in lentiviral infectivity and integration targeting. PLoS One.

[115] Turlure F, Devroe E, Silver PA, Engelman A (2004). Human cell proteins and human immunodeficiency virus DNA integration. Front. Biosci.

[116] Ciuffi A, Llano M, Poeschla E, Hoffmann C, Leipzig J, Shinn P, Ecker JR, Bushman F (2005). A role for LEDGF/p75 in targeting HIV DNA integration. Nat. Med.

[117] Llano M, Vanegas M, Fregoso O, Saenz D, Chung S, Peretz M, Poeschla EM (2004). LEDGF/p75 determines cellular trafficking of diverse lentiviral but not murine oncoretroviral integrase proteins and is a component of functional lentiviral preintegration complexes. J. Virol.

[118] Hao L, Sakurai A, Watanabe T, Sorensen E, Nidom CA, Newton MA, Ahlquist P, Kawaoka Y (2008). Drosophila RNAi screen identifies host genes important for influenza virus replication. Nature.

[119] Brass AL, Huang IC, Benita Y, John SP, Krishnan MN, Feeley EM, Ryan BJ, Weyer JL, van der Weyden L, Fikrig E (2009). The IFITM proteins mediate cellular resistance to influenza A H1N1 virus, West Nile virus, and dengue virus. Cell.

[120] Shapira SD, Gat-Viks I, Shum BO, Dricot A, de Grace MM, Wu L, Gupta PB, Hao T, Silver SJ, Root DE (2009). A physical and regulatory map of host-influenza interactions reveals pathways in H1N1 infection. Cell.

[121] Josset L, Textoris J, Loriod B, Ferraris O, Moules V, Lina B, N’Guyen C, Diaz JJ, Rosa-Calatrava M (2010). Gene expression signature-based screening identifies new broadly effective influenza a antivirals. PLoS One.

[122] Coombs KM, Berard A, Xu W, Krokhin O, Meng X, Cortens JP, Kobasa D, Wilkins J, Brown EG (2010). Quantitative proteomic analyses of influenza virus-infected cultured human lung cells. J. Virol.

[123] Watanabe T, Watanabe S, Kawaoka Y (2010). Cellular networks involved in the influenza virus life cycle. Cell Host Microbe.

[124] Min JY, Subbarao K (2010). Cellular targets for influenza drugs. Nat. Biotechnol.

[125] Chase G, Deng T, Fodor E, Leung BW, Mayer D, Schwemmle M, Brownlee G (2008). Hsp90 inhibitors reduce influenza virus replication in cell culture. Virology.

[126] Gonzalez O, Fontanes V, Raychaudhuri S, Loo R, Loo J, Arumugaswami V, Sun R, Dasgupta A, French SW (2009). The heat shock protein inhibitor Quercetin attenuates hepatitis C virus production. Hepatology.

[127] Soh D, Dong D, Guo Y, Wong L (2010). Consistency, comprehensiveness, and compatibility of pathway databases. BMC Bioinformatics.

[128] Ingenuity Systems http://www.ingenuity.com/index.html.

[129] Konig R, Stertz S, Zhou Y, Inoue A, Hoffmann HH, Bhattacharyya S, Alamares JG, Tscherne DM, Ortigoza MB, Liang Y, Gao Q, Andrews SE, Bandyopadhyay S, De Jesus P, Tu BP, Pache L, Shih C, Orth A, Bonamy G, Miraglia L, Ideker T, Garcia-Sastre A, Young JA, Palese P, Shaw ML, Chanda SK (2010). Human host factors required for influenza virus replication. Nature.

[130] Karlas A, Machuy N, Shin Y, Pleissner KP, Artarini A, Heuer D, Becker D, Khalil H, Ogilvie LA, Hess S, Maurer AP, Muller E, Wolff T, Rudel T, Meyer TF (2010). Genomewide RNAi screen identifies human host factors crucial for influenza virus replication. Nature.

[131] Brass AL, Huang IC, Benita Y, John SP, Krishnan MN, Feeley EM, Ryan BJ, Weyer JL, van der Weyden L, Fikrig E, Adams DJ, Xavier RJ, Farzan M, Elledge SJ (2009). The IFITM proteins mediate cellular resistance to influenza A H1N1 virus, West Nile virus, and dengue virus. Cell.

[132] Shapira SD, Gat-Viks I, Shum BO, Dricot A, de Grace MM, Wu L, Gupta PB, Hao T, Silver SJ, Root DE, Hill DE, Regev A, Hacohen N (2009). A physical and regulatory map of host-influenza interactions reveals pathways in H1N1 infection. Cell.

[133] Josset L, Textoris J, Loriod B, Ferraris O, Moules V, Lina B, N’Guyen C, Diaz JJ, Rosa-Calatrava M (2010). Gene expression signature-based screening identifies new broadly effective influenza a antivirals. PLoS One.

[134] Chen YL, Chen WW, Wang YF, Li RL, Guo WF, Lao SX, Wang JH, Huang SP (2009). Bioinformatics research on chronic superficial gastritis of Pi-deficiency syndrome by gene arrays. Chin. J. Integr. Med.

